# Effect of expanded polytetrafluoroethylene thickness on paclitaxel release and edge stenosis in stent graft

**DOI:** 10.3389/fbioe.2022.972466

**Published:** 2022-07-22

**Authors:** Qing Zhu, Ping Ye, Haifeng Niu, Zhaohua Chang

**Affiliations:** ^1^ Shanghai Institute for Minimally Invasive Therapy, School of Medical Instrument and Food Engineering, University of Shanghai for Science and Technology, Shanghai, China; ^2^ Shanghai MicroPort Endovascular MedTech (group) Co., Ltd, Shanghai, China

**Keywords:** expanded polytetrafluoroethylene, stent grafts, edge stenosis, restenosis, paclitaxel

## Abstract

Stent grafts have been widely used to treat lower extremity arterial stenosis or occlusion. However, there are major issues with edge stenosis and loss of patency over time. Paclitaxel-coated stent grafts have been proven to be effective in preventing edge stenosis, but the insufficient amounts of paclitaxel released may limit the effectiveness of drug-eluting stent grafts. In this study, we examined whether paclitaxel-coated expanded polytetrafluoroethylene (ePTFE) stent graft thickness influences paclitaxel release properties and inhibits edge stenosis. Low-, medium-, and high-thickness paclitaxel-coated stent grafts were prepared by varying the thickness of inner and outer ePTFE layers. Surface morphologies of the stent grafts were analyzed using a scanning electron microscope. The stent grafts were then implanted in the iliac arteries of 20 healthy swine. Twelve pigs were used to assess edge stenosis, and digital subtraction angiography was performed at day 30 (n = 4), 90 (n = 4), and 180 (n = 4). Histological evaluation of the treated arteries was also performed. Eight pigs were used for pharmacokinetic analysis, and the treated arteries were obtained at day 1 (n = 2), 30 (n = 2), 90 (n = 2) and 180 (n = 2). Scanning electron microscopy confirmed that the mean pore size of the stent grafts decreased with increasing thickness. The results of angiographic and histological evaluation demonstrated that low-thickness ePTFE-stent grafts resulted in edge stenosis and apparent intimal hyperplasia at 180 days, whereas for medium-thickness ePTFE-stent grafts, no obvious edge stenosis and intimal hyperplasia was noted in the similar time period. The results of pharmacokinetic evaluation showed that at 180 days, the paclitaxel concentration of treated arteries of the medium group was 36 ± 53 ng/g, while concentrations in the low group was not detectable. Stent grafts with increased ePTFE thickness appear to allow for more delayed release of paclitaxel compared to low-thickness ePTFEs.

## Introduction

The prevalence of lower extremity arterial stenosis occlusive disease is increasing as the population ages. A similar increase in the use of endovascular stents has resulted in a gradual increase in in-stent restenosis. Stenosis greater than 50% of the vessel lumen diameter is considered significant and studies have shown that, 1 year after artery stenting, stenosis was found in 18–40% of patients (Schillinger et al., 2006; Laird et al., 2010). The occurrence of in-stent restenosis is related mainly to the proliferation of smooth muscle cells with neointimal formation (Kim and Dean, 2011). Stent grafts, which include a self-expanding nitinol stent and expanded polytetrafluoroethylene (ePTFE), show better patency rates and long-term outcomes than bare-metal stents (Geraghty et al., 2013; Saxon et al., 2013; Lammer et al., 2015). This suggests that the ePTFE-stent graft may be beneficial for preventing ingrowth of neointimal hyperplasia (Kedora et al., 2007; Saxon et al., 2008; Lammer et al., 2013; Zhang et al., 2015). Nonetheless, neointimal hyperplasia often develops at the edges of the stent graft, and 3-year primary patency rates do not appear to be significantly different between stent grafts and bare nitinol stents (Laird et al., 2012).

Paclitaxel, a commonly used pharmacological inhibitor of neointimal hyperplasia, can be rapidly absorbed by cells (Straubinger et al., 1993), and prevent growth factor–stimulated vascular smooth muscle cell (SMC) migration and proliferation (Axel et al., 1997; Heldman et al., 2001; Sollott et al., 1995). The introduction of paclitaxel to the stent graft leads to effective inhibition of neointimal hyperplasia. In a previous study, paclitaxel was applied to the expanding edge of a balloon during stent graft implantation, and the incidence of edge stenosis was reduced compared with the stent graft no treatment group (Lin et al., 2018). Paclitaxel-coated balloons are significantly effective in preventing edge stenosis, proliferation and migration of SMCs, and extracellular matrix formation leading to neointimal hyperplasia, in stent grafts (Weintraub, 2007). Therefore, the sustained release of paclitaxel may be more effective in inhibiting edge stenosis (Baek et al., 2012; Baek et al., 2015).

The ePTFE is a special porous, biologically and chemically stable material, which makes it a very suitable vehicle for drug transport. According to a previous study, paclitaxel released from an ePTFE polymer matrix in a controlled manner, initially displays a burst release pattern, followed by sustained release due to the rough surface of the ePTFE graft (Lee et al., 2006). We explored the effects of long-lasting paclitaxel drug release on inhibiting edge stenosis in our previous study and proved that the amount of paclitaxel coating and the release pattern have important effects on controlling drug toxicity and neointimal hyperplasia (Zhu et al., 2021). In this study, we used varying thicknesses of ePTFE-stent grafts prepared by spraying paclitaxel, 5 mm on both ends of the stent graft. The paclitaxel release pattern and effects of graft thickness on edge stenosis were then systematically investigated in a swine model.

## Materials and methods

### Materials

Paclitaxel was obtained from Sigma–Aldrich. Covered ePTFE stents, 60 and 30 μm in thickness, were obtained from Hangzhou Anow Microfiltration Co., Ltd. Self-expanding nitinol stents were supplied by Shanghai MicroPort Endovascular MedTech. All other chemicals and reagents used in this study were analytical grade.

### Preparations and characterizations of different thickness ePTFE-stent grafts

The low-, medium-, and high-thickness ePTFE-stent grafts, categorized as low, medium, and high groups for convenience, were manufactured by placing the stent between two ePTFE layers. First, an inner ePTFE layer was placed, then the stent, followed by an outer ePTFE layer, and a fluorinated ethylene propylene (FEP) heat shrinkable sleeve was then applied on the mandrel. The thickness of the applied inner and outer ePTFE layers are shown in Table 1. Then, the entire mandrel assembly was placed into an oven at 350 ± 10°C. After 4 minutes, the mandrel assembly was taken out and subsequently cooled to room temperature. Finally, the FEP heat shrinkable sleeve was removed and the mandrel was pulled out. Both ePTFE layers should have adhered together. The graft thickness was measured by digital thickness gauge.

**TABLE 1:** The ePTFE thickness and the pore size of the low, medium, and high groups.

	Parameters of the ePTFE		ePTFE-stent grafts	
	Inner ePTFE (μm)	Outer ePTFE (μm)	Graft thickness (μm)	Pore size of stent grafts (μm)
Low group	30	30	39.1 ± 10.3	2.8 ± 1.1
Medium group	30	60	50.4 ± 15.1	1.1 ± 0.3
High group	60	60	60.3 ± 13.4	0.6 ± 0.2

The surface morphologies of different graft thicknesses were investigated using a scanning electron microscope (SEM) (FEI, Hillsboro, Ore). The average pore size and surface roughness were evaluated according to SEM results. The average pore size was expressed as the length of the microfibers according to the method described in the literature (Zhang et al., 2004). Microfiber length refers to the medium length of interconnecting fibers connecting adjacent nodes. In this study, we selected 50 internodal fibers on each SEM images of the samples and measured the medium length fibers. The average length of the microfibers was applied as the pore size of the stent grafts.

### Paclitaxel coated ePTFE-stent grafts with varying thicknesses

A drug spray machine was used to coat the drug solution onto the outer surfaces of the stent graft (5 mm long on both ends). Paclitaxel was dissolved in acetone to prepare the drug solution. The stent grafts were mounted at the end of a cylindrical PTFE liner, and the rotation and translation for the PTFE liners were controlled using a software system. The drug solution was fed through a syringe pump. The concentration of drug solution and spraying parameters were uniform for all stent grafts. The designed paclitaxel drug density was 0.3 μg/mm^2^ in terms of stent graft surface area. The actual paclitaxel dose was determined by high performance liquid chromatography (HPLC).

### *In vitro* paclitaxel release pattern of ePTFE-stent grafts with varying thicknesses

In order to investigate the *in vitro* paclitaxel release pattern, a 3% Tween, 80% phosphate-buffered saline (PBS) was used as the release medium. A brown vial was filled with 10 ml of releasing medium, then ePTFE-stent grafts, with varying thicknesses, coated with paclitaxel were placed inside the brown vials, and shaken, at 60 ± 10 rpm, in a shaker at 37°C. At a predetermined time, the release medium was removed and replenished with an equal volume of corresponding fresh release medium. The total paclitaxel amount in the released medium for the stent graft was determined by HPLC using the standard calibration.

### Experimental animals

To evaluate effectiveness of the prepared ePTFE-stent grafts in preventing edge stenosis *in vivo*, we implanted the stent grafts into the left and right iliac arteries of each porcine subject under digital subtraction angiography (DSA) guidance. In this study, the size of the sheath for delivery of all group stent grafts is 7 Fr. The stent grafts have lengths of 40 mm and diameters of 7–10 mm. The average diameter of the porcine iliac arteries is 5.6–9.5 mm. After stent graft implantation, balloon angioplasty was done. The balloon diameter was consistent with the stent graft diameter. The high-thickness group did not feature in our animal experiments because the resistance between the stent graft and the delivery system was too large to release; more details are shown in the discussion section. Heparin was used during the operation at a dose of 100 IU/kg. Aspirin was then administered at 100 mg/day before euthanasia.

### Angiographic and histological evaluation

**FIGURE 1:**
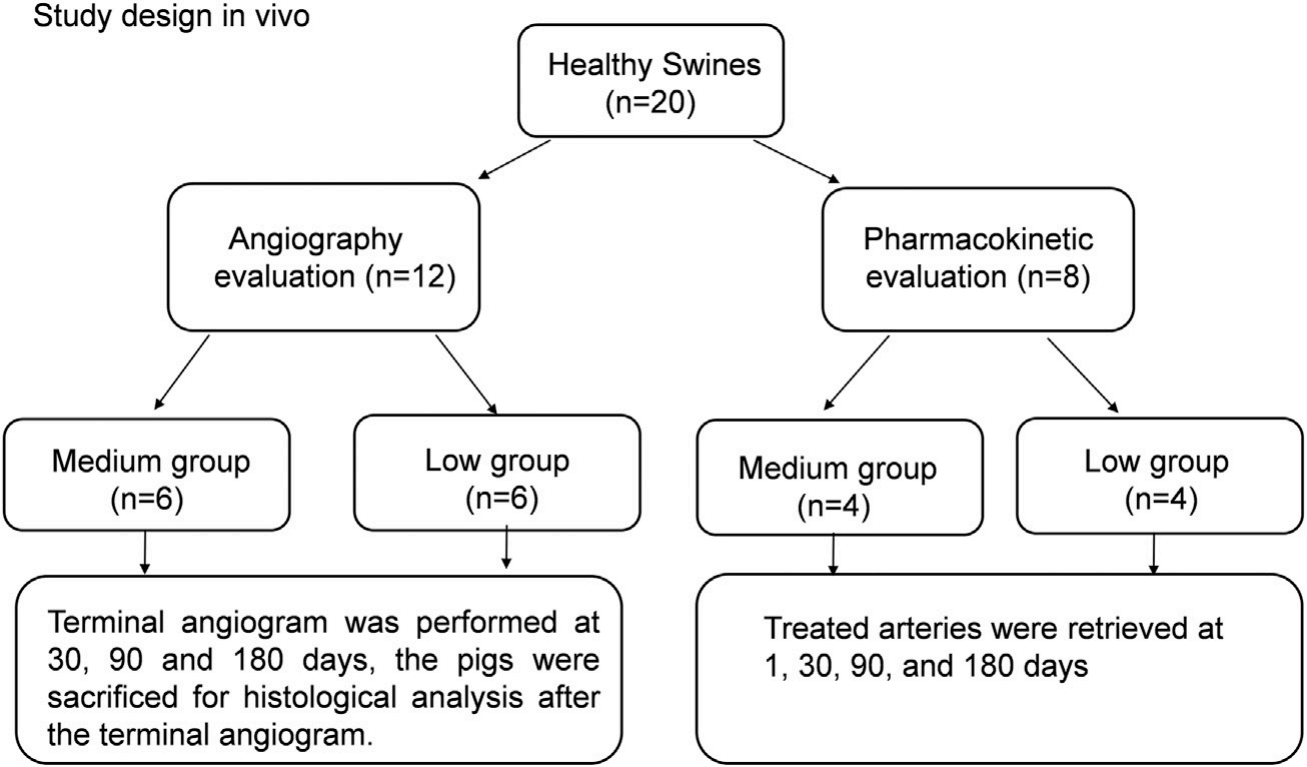
Study design *in vivo*. Angiography evaluation and pharmacokinetic study.

To evaluate edge stenosis after stent implantation, 12 pigs were selected for angiography evaluation for the low-thickness and medium-thickness groups; the grouping details are shown in Figure 1. Six pigs were used for each group. Terminal angiographic evaluation was performed at 30, 90 and 180 days. The implanted stent grafts were taken out along with adjacent blood vessels for histological analysis after terminal angiography.

### *In vivo* pharmacokinetic evaluation

Pharmacokinetic studies were carried out for eight pigs; more details can be seen in Figure 1. Four pigs were selected for each group (the low and medium groups). The treated arteries near the implanted stent grafts were taken out at day 1, 30, 90, and 180. The amount of paclitaxel on the proximal and distal segments of each treated artery was measured by a high-pressure liquid chromatographic-tandem mass spectrometric method (LC-MS).

### Statistical analysis

Experimental data collected in this study are presented as the mean ± standard deviation. One-way analysis of variance was applied to determine statistically significant differences at *p* < 0.05.

## Results

### Preparation and characterization of paclitaxel-coated ePTFE-stent grafts of varying thickness

**FIGURE 2:**
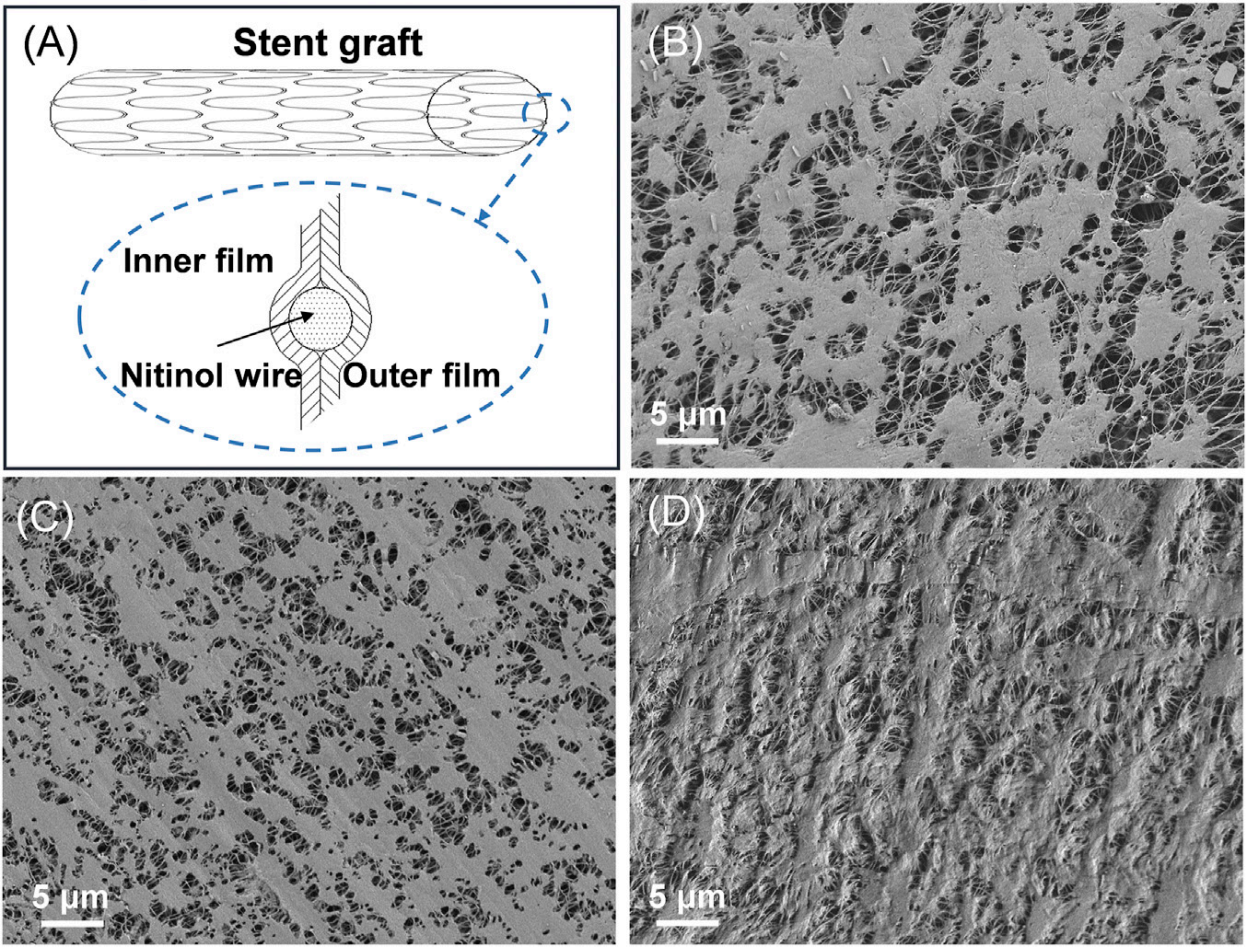
**(A)** Schematic diagram of the ePTFE-stent grafts, SEM images of the ePTFE-stent grafts, the **(B)** low-thickness group **(C)** medium-thickness group, and **(D)** high-thickness group.

The surface morphologies of the ePTFE-stent grafts with varying thicknesses were prepared and characterized by SEM (Table 1; Figure 2, and Supplementary Figure S1). The graft thickness was obtained by adjusting the thickness of the inner and outer ePTFEs, and the average thickness of ePTFE-stent grafts measured by digital thickness gauge was 39.1 ± 10.3, 50.4 ± 15.1 and 60.3 ± 13.4 μm for the low, medium, and high groups, respectively. As shown in Figures 2A–D, stent grafts with different graft thicknesses showed significant difference in pore size and surface roughness. In this study, we selected 50 internodal fibers and measured the medium length fibers on each SEM image of the samples (Figures 2B–D). The average length of the microfibers was applied as the pore size of the stent grafts. The calculated average pore size was 2.8 ± 1.1, 1.3 ± 0.3, and 0.6 ± 0.2 μm for the low, medium, and high groups. Surface roughness was also evaluated according to SEM results, as shown in Supplementary Figure S1. It can be seen from the images that the roughness of the high-thickness group (Supplementary Figure S1C) is significantly increased than those of the medium- and low-thickness groups (Supplementary Figures S1A,B).

### *In vitro* paclitaxel release

**FIGURE 3:**
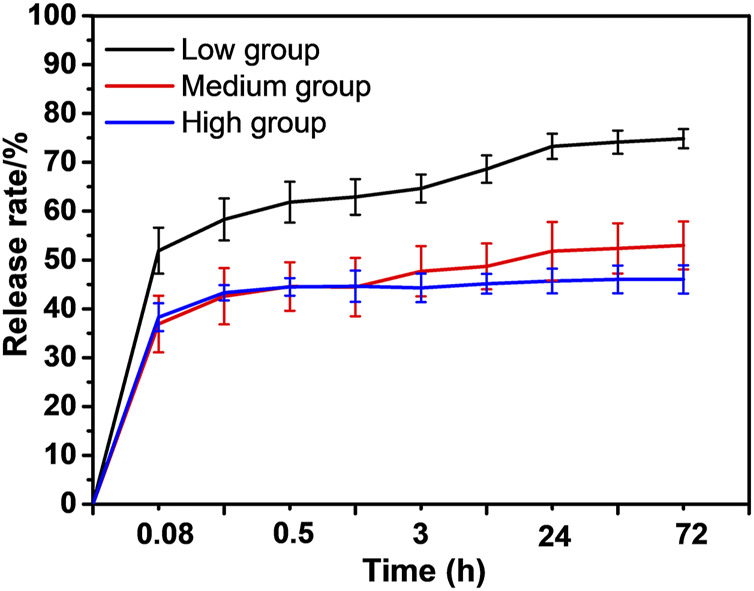
The *in vitro* paclitaxel release pattern of the experimental groups.

The drug loading amount (0.3 μg/mm^2^) and *in vitro* paclitaxel-release patterns of the ePTFE-stent grafts were characterized by the HPLC. The actual paclitaxel loading amount was statistically the same for the low, medium, and high groups, at 0.32 ± 0.11, 0.31 ± 0.07 and 0.33 ± 0.09 μg/mm^2^, respectively, as shown in Supplementary Figure S2. The ePTFE-stent grafts with varying thicknesses showed various *in vitro* paclitaxel release patterns, as shown in Figure 3. The low-thickness group showed the highest *in vitro* paclitaxel release pattern and its cumulative release amount reached (74.8 ± 4.9%) at 72 h. The medium- and high-thickness groups exhibited slow paclitaxel release patterns; the initial release amounts of paclitaxel were less than 40%, and the cumulative release amounts were (51.9 ± 5.7%) and (46.8 ± 4.2%) at 72 h, respectively.

### Angiographic and histological evaluation

**FIGURE 4:**
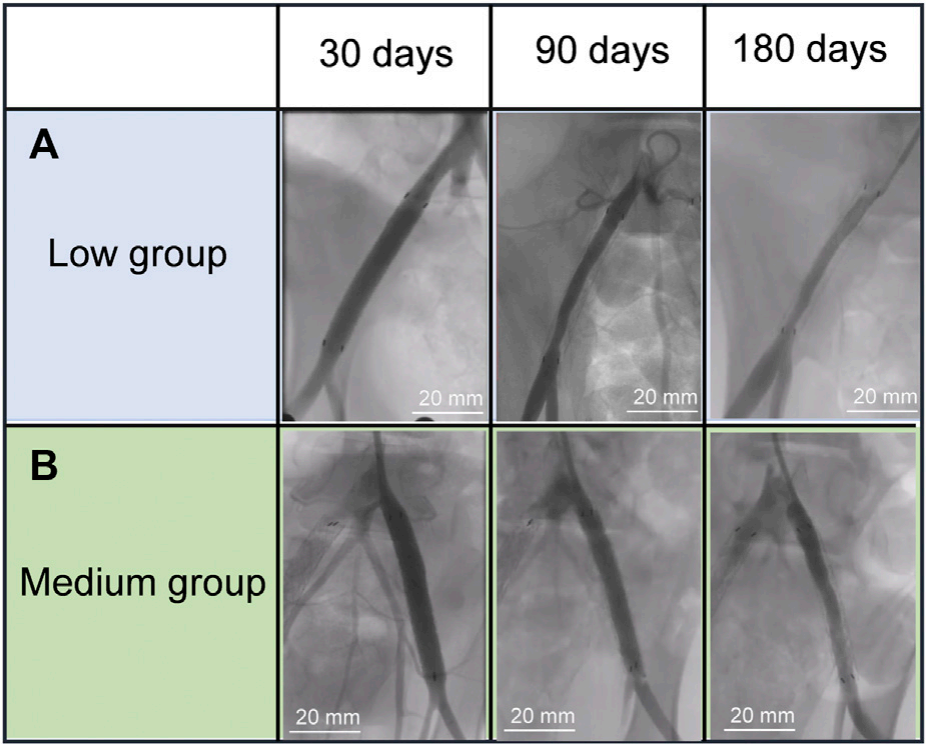
Angiography evolution of the stent grafts in the swine iliac artery **(A)** low group, **(B)** mediun group.

To study the edge stenosis on paclitaxel-coated ePTFE-stent grafts of varying thicknesses *in vivo*, angiographic evaluations of all stent grafts were performed at 30, 90, and 180 days after implantation by DSA (Figure 4.) For the paclitaxel-coated low group (Figure 4A), slight stenosis at the distal end of the stent graft was found at 90 days, and apparent edge stenosis was discovered at the proximal and distal edges of the stent graft at day 180. In contrast, no apparent edge stenosis was discovered in the medium group at 180 days, as shown in Figure 4B.

**FIGURE 5:**
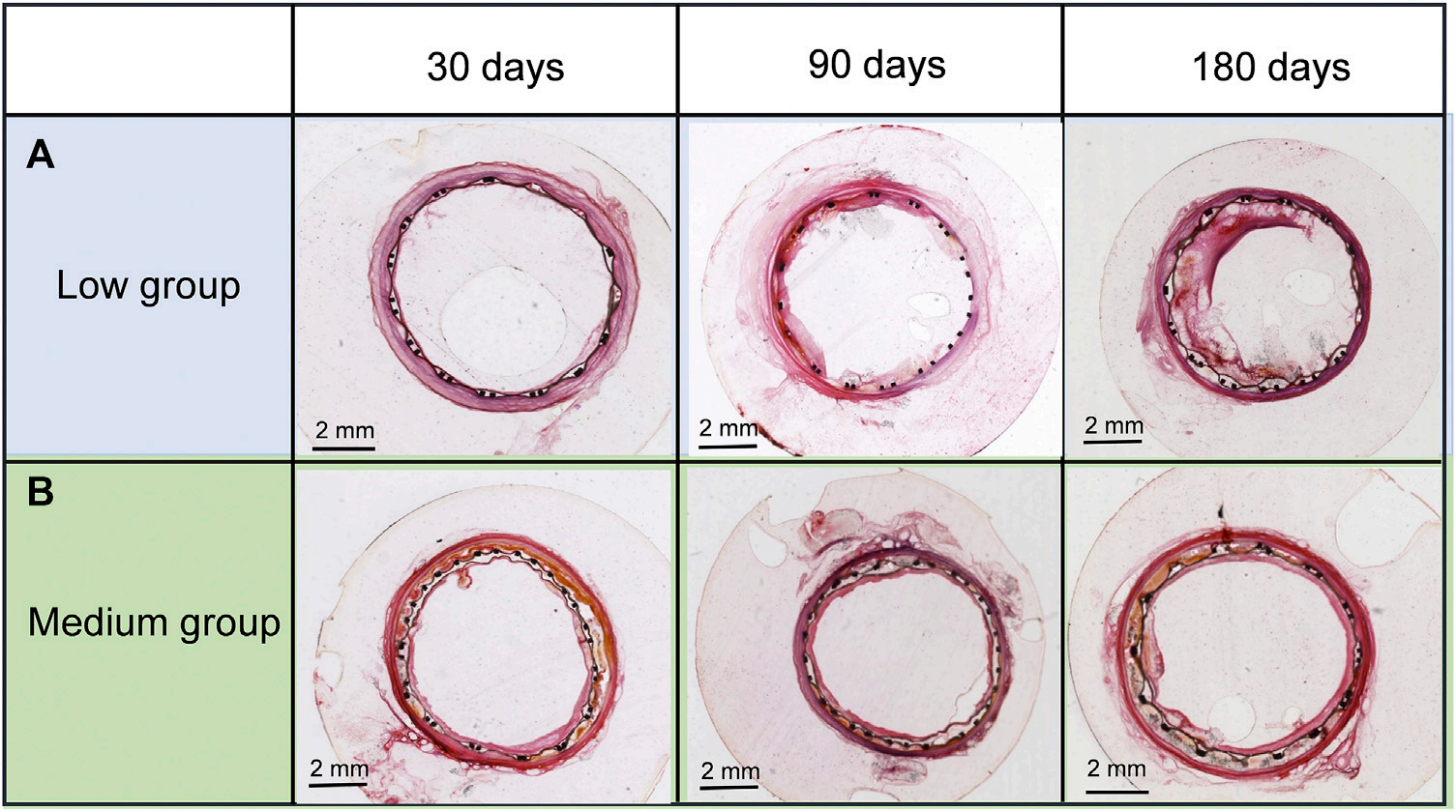
Cross-sections of **(A)** the low- and **(B)** medium-thickness ePTFE-stent grafts at 30, 90, and 180 days. Specimens were stained with hematoxylin and eosin.

Treated arterial cross-sections of the low and medium groups at 30, 90, and 180 days are shown in Figure 5. In the low group (Figure 5A), no evident intimal hyperplasia of stent grafts was found at 30 days. Slight intimal hyperplasia of stent grafts was seen at 90 days and apparent intimal hyperplasia manifested at 180 days within the denatured outer and interior cells membranes. For the medium group (Figure 5B), no evident intimal hyperplasia of stent grafts was found at 30 and 90 days, although slight intimal hyperplasia was observed at 180 days. These results indicated the medium-thickness group showed significantly less neointimal formation than the low-thickness group.

### *In vivo* pharmacokinetic evaluation

To reveal the long-term release of paclitaxel *in vivo*, four pigs were selected for the low and medium groups, respectively. The paclitaxel concentrations of the stent grafts in the arteries at day 1, 30, 90, and 180 were detected by LC-MS and shown in Table 2. For the low group, a paclitaxel burst (33,465 ± 2,768 ng/g) was found in the treated artery at day one, decreasing to 51 ± 39 ng/g at 90 days, and was undetectable at 180 days. For the medium group, the treated arterial paclitaxel concentration decreased from 14,682 ± 1,568 at 1 day to 36 ± 53 ng/g at 180 days. Paclitaxel release in the medium-thickness group *in vivo* showed a longer-lasting effect than that in the low-thickness group.

**TABLE 2:** Paclitaxel concentration in the treated arteries.

Time	Low group (ng/g)	Medium group (ng/g)
Day 1	33,465 ± 2,768	14,682 ± 1,568
Day 30	970 ± 380	1,632 ± 568
Day 90	51 ± 39	134 ± 78
Day 180	below the quantification limit	36 ± 53

## Discussion

Paclitaxel inhibits smooth muscle cell proliferation and migration by stabilizing microtubules, thus preventing neointimal hyperplasia (Straubinger et al., 1993; Sollott et al., 1995; Axel et al., 1997; Banerjee et al., 2015; Heldman et al., 2001). In our previous study, severe edge stenosis was observed at the proximal and distal edges of the ePTFE-stent grafts without paclitaxel coating, while stents coated with paclitaxel (5 mm long at each end) played a positive role in inhibiting stent edge stenosis. Paclitaxel release patterns affected the effectiveness of coated stent grafts (Zhu et al., 2021). The controlled release pattern of drug molecules, achieved by modifying a polymer matrix, is the basis of many controlled release methods. A stent graft is an effective combination of bare stent and ePTFEs, which are biocompatible, chemically inert, and modifiable medical materials. In this study, varying thicknesses of ePTFE-stent grafts were prepared and sprayed with paclitaxel at their distal ends (5 mm from each end). The release pattern of paclitaxel from the stent graft was controlled by modifying graft thickness.

According to the SEM results, stent grafts with different ePTFE thicknesses have different surface morphologies, which are mainly reflected in the obvious differences in the pore size and surface roughness of the stent grafts. With an increase in graft thickness, the pore size of the stent grafts decreases, while the stent surface roughness increases. The high-thickness group was not included in our animal experiments because of the great resistance between the stent graft and the delivery system, which made it difficult to release paclitaxel. Through the analysis of SEM images, we discovered two main reasons for the excessive resistance. One is the surface of the high-thickness stent graft is too rough, and this surface roughness was observed in SEM images of grafts with varying thicknesses. Another reason is that although graft thickness has only increased by 10 μm, the volume of the graft increased by 20%. When the stent graft was assembled into a 7 Fr delivery system, the resulting profile of the stent graft after compression was much larger, resulting in a mismatch between the profile of the compressed stent graft and the inner diameter of the delivery system.

In investigating the vitro release of paclitaxel, the increased graft thickness was useful at slowing down the initial release rate, and the high-thickness group only showed a slight decrease in cumulative release amounts of paclitaxel than that of the medium-thickness group. This indicates that graft thickness affects the *in vitro* paclitaxel release pattern; the appropriate increase in thickness helps reduce the *in vitro* paclitaxel release rate. Drugs are generally released from polymer systems through two physical processes in this study, namely surface release (dissolution of the drug into the release medium) and intramembrane release (diffusion of the drug from the polymeric matrix), denoting that graft thickness has a certain influence on drug release patterns (Acharya and Park, 2006; Raval et al., 2010). For the low-thickness group, drug release was primarily regulated by dissolution due to the low graft thickness and large pore size of the stent graft. For the medium- and high-thickness groups, drug release was primarily controlled by slow diffusion as the release medium could not easily penetrate the high-thickness and small pore size ePTFEs. Therefore, the paclitaxel release rate decreases with increasing graft thickness, and the high-thickness group only showed a slight decrease in cumulative amounts of paclitaxel release than that in the medium-thickness group. This indicates that graft thickness affects *in vitro* paclitaxel release pattern; the appropriate increase in thickness helps to reduce the *in vitro* paclitaxel release rate.

According to previous studies, neointimal proliferation mainly occurs at the edges of stent grafts (Geraghty et al., 2013). A long-lasting drug effect from stent grafts after implantation is essential to inhibit neointimal hyperplasia from proliferation and migration of SMCs and extracellular matrix formation. In this study, the medium-thickness group more effectively demonstrated the suppression of edge stenosis, as seen from angiographic evaluations, with less neointimal formation in treated arterial cross-sections, than the low-thickness group. Arterial paclitaxel concentrations in the medium-thickness group was 36 ± 53 ng/g at 180 days, which is still within the therapeutic range of paclitaxel (Raval et al., 2010). The medium-thickness group exhibited a lower vivo paclitaxel release rate and longer release effect than the low-thickness group, which was consistent with the results of paclitaxel release *in vitro*. In summary, an increase in graft thickness can prevent intimal hyperplasia and decelerate paclitaxel release rate. In conjunction with this, the medium-thickness group showed a longer-lasting effect in inhibiting edge stenosis than the low-thickness group. This may be due to the observation that medium-thickness ePTFEs have smaller pores, which is conducive to the slow release of paclitaxel and can inhibit intimal hyperplasia for a long time. Dolmatch et al. demonstrated that, compared to bare stents in a porcine model, the porosity of the stent-graft at 6 weeks after implantation can limit neointimal tissue proliferation, which was attributed to faster endothelialization (Dolmatch et al., 2007). However, in clinical settings, the stent graft produces intimal hyperplasia resulting in edge stenosis within 1 year after implantation, and the average mean duration before stenosis developed was 10.7 months (Golchehr et al., 2015). Based on our experimental data, the continuous release of paclitaxel for 6 months could be achieved by adjusting the graft thickness of stent grafts, which could then inhibit intimal hyperplasia in porcine iliac arteries. This suggests that our stents can inhibit intimal hyperplasia and reduce the occurrence of edge stenosis by means of controlling the release patterns of paclitaxel.

However, this study still has some limitations. For example, only three graft thicknesses of ePTFE-stent grafts were designed and investigated in this study, more varied graft thicknesses should be considered for investigation in the future. Another limitation was that this study was performed in the healthy porcine model and cannot completely simulate atherosclerotic conditions in humans. Additionally, the animal sample size used in this study was small due to constraints on funding, so that more experimental animals should be considered in a later study. Furthermore, comparative experimentation between our prepared stent grafts and commercial stent grafts should also be considered in the future.

## Conclusion

In this study, ePTFE-stent grafts of varying thicknesses were prepared and coated with paclitaxel on their outer surfaces (5 mm from each end). Subsequently, the paclitaxel release pattern of the stent grafts were investigated systematically. The results showed that medium- and high-thickness ePTFE-stent grafts showed long-lasting *in vitro* paclitaxel release patterns than the low-thickness ePTFE-stent grafts. The medium-thickness ePTFE-stent grafts were more effective in reducing edge stenosis, *in vivo,* at 180 days than the low-thickness ePTFE-stent grafts, and the edge stenosis-suppressing effects of the *in vivo* paclitaxel release can be extended up to 180 days. The study results indicate that the ePTFE-stent grafts with varying graft thicknesses exert an important influence on paclitaxel release pattern and inhibition of edge stenosis.

## Data Availability

The raw data supporting the conclusions of this article will be made available by the authors, without undue reservation.
