# Supplementary material for: Effect of expanded polytetrafluoroethylene thickness on paclitaxel release and edge stenosis in stent graft
Source: Front Bioeng Biotechnol. 2022 Jul 22;10:972466. doi: 10.3389/fbioe.2022.972466 (PMC9354930; doi:10.3389/fbioe.2022.972466)
Supplement: Supplementary file 1 [file DataSheet1.pdf]

Effect of expanded polytetrafluoroethylene thickness on paclitaxel release and edge stenosis in stent graft

Qing Zhu[1,2], Ping Ye[1], Haifeng Niu[2], Zhaohua Chang[1,2*]

[1]Shanghai Institute for Minimally Invasive Therapy, School of Medical Instrument and Food Engineering, University of Shanghai for Science and Technology, Shanghai, PR China.

[2]Shanghai MicroPort Endovascular MedTech (group) Co., Ltd, Shanghai, PR China.

*** Correspondence:**
Zhaohua Chang
m8090313@126.com

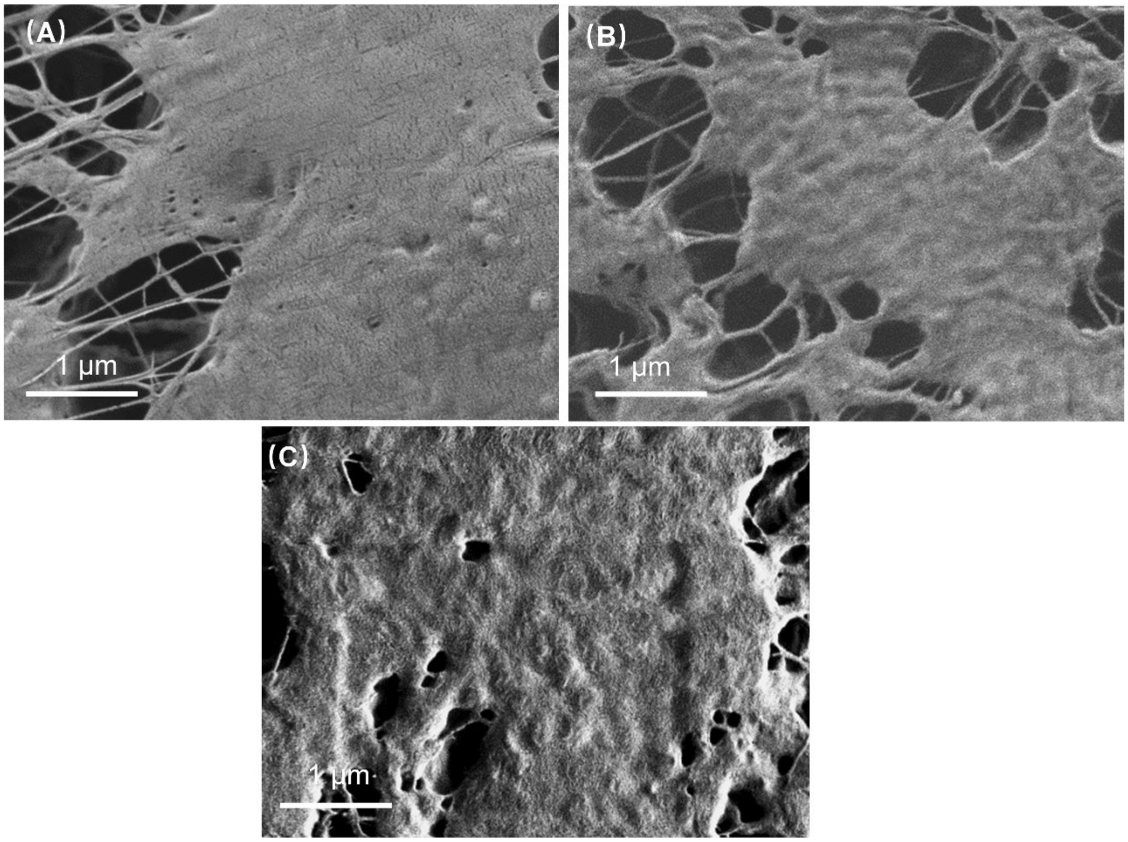

Figure S1. SEM images of the ePTFE-stent grafts, (A) low group (B) medium group and (C) high group

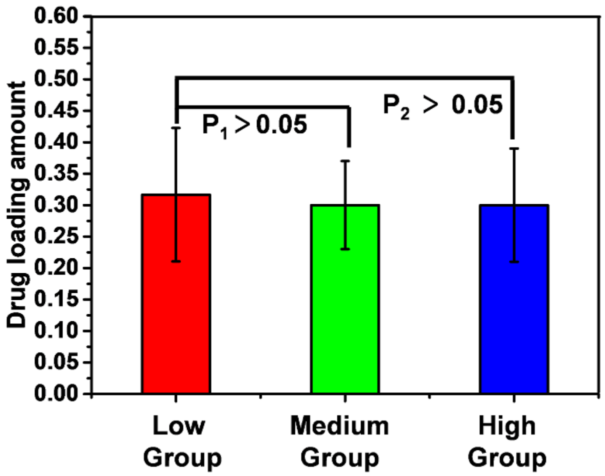

Figure S2. The actual paclitaxel loading amount of the stent grafts
